# *Fusobacterium nucleatum* upregulates the immune inhibitory receptor *PD-L1* in colorectal cancer cells via the activation of ALPK1

**DOI:** 10.1080/19490976.2025.2458203

**Published:** 2025-01-29

**Authors:** Coco Duizer, Moniek Salomons, Merel van Gogh, Sanne Gräve, Freke A. Schaafsma, Maaike J. Stok, Merel Sijbranda, Raghuvandhanan Kumarasamy Sivasamy, Rob J. L. Willems, Marcel R. de Zoete

**Affiliations:** Department of Medical Microbiology, University Medical Center Utrecht, Utrecht, The Netherlands

**Keywords:** *Fusobacterium nucleatum*, ADP-heptose, ALPK1, *PD-L1*, colorectal cancer, immune checkpoint inhibitor therapy, Fusobacterium, DNA damage response

## Abstract

*Fusobacterium nucleatum* is a Gram-negative oncobacterium that is associated with colorectal cancer. The molecular mechanisms utilized by *F. nucleatum* to promote colorectal tumor development have largely focused on adhesin-mediated binding to the tumor tissue and on the pro-inflammatory capacity of *F. nucleatum*. However, the exact manner in which *F. nucleatum* promotes inflammation in the tumor microenvironment and subsequent tumor promotion remains underexplored. Here, we show that both living *F. nucleatum* and sterile *F. nucleatum-*conditioned medium promote CXCL8 release from the intestinal adenocarcinoma HT-29 cell line. We determined that the observed pro-inflammatory effect was ALPK1-dependent in both HEK293 and HT-29 cells and that the released *F. nucleatum* molecule had characteristics that match those of the pro-inflammatory ALPK1 ligand ADP-heptose or related heptose phosphates. In addition, we determined that not only *F. nucleatum* promoted an ALPK1-dependent pro-inflammatory environment but also other *Fusobacterium* species such as *F. varium*, *F. necrophorum* and *F. gonidiaformans* generated similar effects, indicating that ADP-heptose or related heptose phosphate secretion is a conserved feature of the *Fusobacterium* genus. By performing transcriptional analysis of ADP-heptose stimulated HT-29 cells, we found several inflammatory and cancer-related pathways to be differentially regulated, including DNA mismatch repair genes and the immune inhibitory receptor *PD-L1*. Finally, we show that stimulation of HT-29 cells with *F. nucleatum* resulted in an ALPK1-dependent upregulation of *PD-L1*. These results aid in our understanding of the mechanisms by which *F. nucleatum* can affect tumor development and therapy and pave the way for future therapeutic approaches.

## Introduction

Colorectal cancer (CRC) poses a significant global health challenge, ranking as the third most prevalent type of cancer worldwide and contributing to two million new cases and one million deaths annually.^1^ While immune checkpoint inhibitor therapy, targeting for instance PD-1/PD-L1, has been shown to be effective in treating CRC, it is often limited to tumors with a microsatellite instability high (MSI-H) phenotype caused by mutations in DNA mismatch repair genes.^2^ *F. nucleatum* has emerged as a prominent bacterial contributor to CRC, with multiple studies revealing its enrichment in or on tumor tissue compared to matched normal tissue.^3–5^ While it is not fully elucidated how *F. nucleatum* contributes to CRC, various mechanisms have been proposed. For instance, it has been shown that the glycan Gal-GalNAc, which is upregulated in CRC tissue, allows for the efficient adherence of the bacterium via its receptor Fap2.^6^ Fap2 has also been implicated in inhibiting NK cells from killing tumor cells by binding its TIGIT receptors.^7^ The *F. nucleatum* adhesin FadA binds to E-cadherin and induces epithelial proliferation.^8^ In addition to its abundance in tumor tissue, *F. nucleatum* is also associated with cancer recurrence and poor patient survival.^9^ By inducing autophagy, *F. nucleatum* promotes chemoresistance, which was shown to be mediated through TLR4 and MyD88-dependent pathways.^9^ Furthermore, *F. nucleatum* abundance correlates with resistance to the tyrosine kinase inhibitor regorafenib and has been shown to increase both the resistance and sensitivity to PD-1 blockade therapy in patients, through the secretion of succinic acid and subsequent impairment of CD-8^+^ T cell immunity^10^ and the upregulation of PD-L1,^11,12^ respectively.

Besides directly influencing tumorigenesis and evasion of anti-tumor immunity, *F. nucleatum* may also contribute to cancer via the induction of (chronic) inflammatory responses, which may play an important role at the early stages of tumor development. Indeed, several studies have reported the pro-inflammatory capabilities of *F. nucleatum*. For instance, the binding of *Fusobacterium* to E-cadherin resulted in pro-inflammatory responses.^8^ In HCT116 colorectal cancer cells, *F. nucleatum* promoted the upregulation of TLR4 and MyD88 and the activation of the major immunological transcription factor NF-κB.^13^ Such inflammatory responses were not limited to *in vitro* models, as *F. nucleatum* induced a pro-inflammatory environment in tumor tissue in infected Apc^min/+^ mice.^3^ Similarly, the presence of *F. nucleatum* correlated with a pro-inflammatory tumor microenvironment in human CRC tissue from the Cancer Genome Atlas (TCGA).^3^

Despite the growing understanding of the role that immunostimulatory responses play in *F. nucleatum*-induced tumorigenesis in *in vitro* and *in vivo* models, the mechanistic link between *F. nucleatum* and the induction of inflammation remains incompletely elucidated. In this study, we set out to delineate immunological pathways through which *F. nucleatum* contributes to CRC development. We reveal that *F. nucleatum* secretes the pro-inflammatory metabolite ADP-heptose (and/or related heptose phosphates), initiating an NF-κB response via alpha-kinase 1 (ALPK1). Transcriptome analysis of ADP-heptose-stimulated colorectal adenocarcinoma cells shows a global pro-inflammatory response, a decreased expression of DNA mismatch repair genes and an increase in *PD-L1* expression. Altogether, our findings expand the comprehension of the role of *F. nucleatum* in CRC development and open avenues for targeted therapeutic interventions in *F. nucleatum*-positive colorectal cancer.

## Material & methods

### Cell culture

HEK293, HEK293 *ALPK1*^−/−^, HT-29 and HT-29 *ALPK1*^−/−^ cell lines were routinely cultured in Dulbecco’s modified Eagle medium (DMEM; Thermo Fisher Scientific) supplemented with 10% fetal bovine serum (FBS; Gibco) under 5% CO_2_ at 37°C unless stated otherwise. Human-derived rectal organoid cell line Pt15–70206 was grown according to the protocol described previously.^14^ In short, rectal organoids were cultured in domes of BME (Cultrex RGF BME, type 2) covered by medium containing 15% Advanced DMEM/F12, 1× Glutamax, 100 U/mL Penicillin- Streptomycin, 10 mmol/L HEPES (all Invitrogen), 25% Rspo1 conditioned medium, 10% Noggin conditioned medium, 50% Wnt conditioned medium, 2% B27 (Invitrogen), 10 mm Nicotinamide, 1.25 mm N-acetylcysteine, and 3 μM P38 inhibitor (SB202190; all Sigma- Aldrich), 50 ng/mL mEGf (Invitrogen), 500 nM A83–01 (Tocris). Prior to stimulation, organoids were differentiated by adding the same medium without Wnt conditioned medium, Nicotinamide and p38 inhibitor for 5–7 days.

### CRISPR/Cas9-mediated gene editing

HEK293 *ALPK1*^−/−^ cells were generated as previously described.^15^ HT-29 *ALPK1*^−/−^ cells were generated using the CRISPR Gene Knockout Kit v2 (Synthego) according to the manufacturer’s protocol with the following sgRNA sequences, sgRNA 1: 5’-CAUCCUCGCUCGGGACUGUG-3’, sgRNA 2: 5’-CUGUAUGGGCUCGACGUCUC-3’, sgRNA 3: 5’-AGUUCACGGAGAUUCGGGCU-3’. To confirm that the deletion was correct, HT-29 *ALPK1*^−/−^ cells were first analyzed by PCR amplification of the deletion region using ALPK1 fw: 5’- TCTATTTTCTTTCTTTCGGTTCAGC-3’ and ALPK1 rv: 5’- TCCGGGTGTCCCACAGA-3’ followed by Sanger-sequencing (Macrogen) of the purified PCR product using the primer ALPK1 seq: 5’- CTAACAAAGTATTTTTCTCTTCCTAGCTGT-3’. In addition, the loss of a functional ALPK1 was confirmed by analyzing ADP-heptose-stimulated HT-29 *ALPK1*^−/−^ cells using a Human IL-8/CXCL8 DuoSet ELISA DY208 (R&D Systems).

### Bacterial culture

All bacterial strains (Table S1) were grown under anaerobic conditions in an anaerobic chamber (Coy Laboratory Products Inc. 85% N_2_, 10% CO_2_, 5% H_2_). Prior to culturing in broth, bacteria were grown on Gut Microbiota Medium (GMM^16^) agar plates for 2 to 3 days at 37°C. Individual colonies were taken from the plate and used to inoculate starter cultures in GMM broth without Tween-80 and resazurin under anaerobic conditions at 37°C overnight. Next, these cultures were used to inoculate fresh cultures at a starting OD_600_ of 0.05 and grown for 48 h at 37°C. To obtain sterile bacterial conditioned culture medium, 1 mL of the cultures were centrifuged at 10.000 × *g*, after which medium was collected. Media were sterilized using 0.2-μm syringe filters (Corning). To obtain live bacteria for bacteria-host cell co-cultures, the OD_600_ of cultured bacteria was measured, bacteria were centrifuged at 10.000 × *g*, and washed twice with PBS and resuspended in DMEM to reach a final multiplicity of infection (MOI) of 100 after adding 15 μL bacterial suspension to the host cells.

### Fractionation of bacterial conditioned medium

Bacterial media, ligands or controls were heat-inactivated (HI) to denature proteins and determine heat stability by incubation for 10 min at 95°C. To oxidize carbohydrates, bacterial media were treated with sterile sodium metaperiodate (SP, Fisher Scientific) at a final concentration of 10 mm or MilliQ (mock SP) for 1 h at 20°C. Lastly, media were applied to 3,000 and/or 100,000 MWCO kDa centrifugal filter device (Amicon, Ultra) to separate media based on molecular size. The retentate was resuspended in an equal volume of medium.

### Cell stimulations

For ELISA experiments, HT-29, HT-29 *ALPK1*^−/−^ or HEK293 cells were seeded in 96-well plates (Corning). After 24 h, cell culture medium was replaced with 100 μL of DMEM. Cells were stimulated with GMM (medium control), *Fusobacterium* filter-sterilized conditioned medium, treated conditioned medium (as described above), live *Fusobacterium* at an MOI of 100 resuspended in DMEM, DMEM control, 50 ng/mL ADP-L-heptose (InvivoGen) or 10 μg/mL Fla-ST Ultrapure (InvivoGen), all in a volume of 15 μL. Cells were incubated for 24 h at 37°C under 5% CO_2_ and 21% O_2_, or in an hypoxia chamber at an atmosphere of 5% CO_2_, 5% O_2_ and 90% N_2_ when indicated.

For NF-κB luciferase reporter assays, culture medium from transfected HEK293 or HEK293 *ALPK1*^−/−^ cells was replaced with 100 μL DMEM followed by stimulation with 5 μL of GMM medium control, filter-sterilized conditioned media of the indicated bacteria, 400 ng/mL recombinant human TNF (InvivoGen) or 50 ng/mL of ADP-L-heptose for 5.5 h at 37°C.

For RNA expression analysis, organoids, HT-29 and HT-29 *ALPK1*^−/−^ cells were stimulated with 500 ng/mL ADP-L-heptose (InvivoGen), 500 ng/mL Flagellin-ST ultrapure (InvivoGen), 10% v/v of bacterial conditioned medium or GMM, or not-stimulated for 2.5 h (for RNA-SEQ analysis) or various timepoints and ADP-L-heptose concentrations as indicated per experiment. Cells were pre-treated with 5 uM of the NF-κB inhibitor BAY 11–7082 (Sigma-Aldrich). After 30 min, cells were stimulated with 500 ng/ml ADP-L-heptose for 6 h. Cells were washed twice with cold PBS and subsequently lysed in buffer RLT (Qiagen) and stored at −20°C.

### ELISAs

To perform ELISAs, host cell media were collected in V-bottom plates (Greiner), centrifuged at 640 × *g* for 5 min at 20°C, transferred to a new V-bottom plate and stored at −20°C until further use. The IL-8 human uncoated ELISA kit (Invitrogen) was used to assess the concentration of released CXCL8 in thawed cell conditioned media according to the manufacturer’s protocol.

### Transfections

HEK293 or HEK293 *ALPK1*^−/−^ cells were seeded in a 6-well plate at a cell density of ~ 50% and transfected the following day with 1 μg of a NF-κB luciferase reporter plasmid (pNFkB-Luc (V004337#), NovoPro Bioscience) and either 1 μg of an ALPK1 complementation plasmid^15^ or LacZ control plasmid using FuGENE HD Transfection Reagent (Promega). In short, 6 μL FuGENE was mixed with 150 μL DMEM without FBS, mixed and incubated for 10 min at 20°C. 50 μL of DMEM containing 1 μg plasmid DNA was added to the FuGENE/DMEM mixture, vortexed and incubated for 5 min at 20°C. The FuGENE/DNA mixture was then added to the wells and incubated for 24 h. Cells were subsequently trypsinized, seeded in 96-well plates and incubated for another 24 h before stimulations.

### Nf-κB reporter assays

After stimulation, culture medium was aspirated and cells were lysed by mixing with 50 μL of reporter lysis buffer (Promega) and freezing at −80°C for at least an hour. 37 μL of luciferase (Firefly Luciferase Assay System, Promega) was added to 15 μL lysates mixed and immediately measured using a CLARIOstar microplate reader (BMG Labtech) at an emission wavelength of 500–660 nm.

### RNA isolation

Cells were washed twice with cold PBS and subsequently lysed in TRIzol (Invitrogen) or buffer RLT (Qiagen) and stored at −20°C. RNA isolation was performed using the RNA/miRNA Tissue/cells kit with the EZ2 Connect (Qiagen) according to the manufacturer’s protocol, or using TRIzol with the complete RNA isolation protocol for qPCR samples or until the phase separation step for the RNA-sequencing samples. Subsequently, for these samples, the aqueous phase (containing the RNA) was transferred to an RNeasy mini kit (Qiagen) column to continue RNA isolation. Next, the RNeasy RNA isolation protocol was followed from the addition of 70% ethanol onwards. DNA contamination was removed using TURBO DNA-free Kit (Invitrogen) according to protocol. RNA concentrations and purity were measured using the Qubit 2.0 Fluorometer (Invitrogen) and Nanodrop spectrophotometer (Thermo Scientific), respectively.

### RNA-sequencing analysis

RNA sample quality control, mRNA library preparation (poly A enrichment) and sequencing (Illumina NovaSeq PE150) was performed by Novogene. Each sample gave a minimum of 6 GB of raw data with a Q30 ≥ 80%. FastQ files were used for alignment to the human genome using HISAT2^17^ and counts were determined using featureCounts.^18^ Data were analyzed and visualized using RNAseqChef^19^ using DESeq2, a False Discovery Rate of 0.05 using Benjamini-Hochberg correction with a twofold change cutoff. For pathway enrichment analyses in RNAseqChef, KEGG (v. 1.64.1) and MSigDB Hallmark (v. 7.5.1) genesets were used with a False Discovery Rate of 0.05 using Benjamini-Hochberg correction and an onefold change cut-off. Three-conditions differential expression analysis was performed using the empirical Bayesian hierarchical modeling approach EBSeq encoded in RNAseqChef.

### RT-qPCR

RNA concentrations were normalized in ultrapure DNase/RNase free water (Invitrogen). RT-PCR reaction was performed using the Maxima first strand cDNA synthesis kit (ThermoFisher Scientific) according to manufacturer’s protocol. qPCR was performed using the Taqman Fast Advanced Master Mix (ThermoFisher Scientific) and Taqman Gene Expression Assay (FAM) (ThermoFisher Scientific) according to manufacturer’s protocol. The following probes were used: *Actb* (HS01060665), *CD274* (*PD-L1;* HS00204257), *MSH2* (HS00953527), *MSH6* (HS00943000), *MLH1* (HS00979919) and *PMS2* (HS00241053).

### Western blot

HT-29 and HT-29 *ALPK1*^−/−^ cells were seeded in a 12-well plate. After 24 h, medium was replaced with DMEM and cells were either none-stimulated or stimulated with 500 ng/ml ADP-L-heptose. After 0, 5 or 20 min of stimulation, cells were washed twice with ice-cold PBS and lysed with Laemmli sample buffer (Bio-Rad). Proteins were separated on a 4–12% NuPAGE gel (Invitrogen), transferred to a nitrocellulose membrane using the Trans-Blot Turbo Transfer System (Bio-Rad) and blocked in Tris-buffered saline (TBS, 20 mm Tris, 150 mm NaCl, pH 7.6) containing 0.1% Tween-20 and 5% nonfat milk. Proteins were detected using Phospho-IkappaB-alpha (Ser32) antibodies (#9241, Cell Signaling) or anti-beta Actin antibodies (ab8227, Abcam).

### Flow cytometry

24 h after seeding HT-29 and HT-29 *ALPK1*^−/−^ cells in 48-well plates (Corning), cells were stained with 5 uM CellTraceTM CFSE (Invitrogen) for 20 min at 37°C, according to the manufacturer’s protocol. Cells were subsequently stimulated with 500 ng/ml ADP-L-heptose in DMEM. Proliferation was assessed by flow cytometry after 24, 48 and 72 h using the FACSCanto (BD). FlowJoTM v10.8.1 software (BD Life Sciences) was used to analyze the median fluorescent intensity (MFI).

### Statistical analysis

All statistical analyses were performed using GraphPad Prism version 10.1.1. Where appropriate significance was calculated using a (non-)paired Student t-test or a ratio paired t test, as indicated in the figure legends. For qPCR data, statistical testing was performed on dCT values. Three-condition differential expression analysis was performed using the empirical Bayesian hierarchical modeling approach EBSeq encoded in RNAseqChef. Significance was annotated with asterisk; *p* value 0.01–0.05 = *, 0.001–0.01 = **, <0.001 = ***, not significant=ns.

## Results

### *Fusobacterium nucleatum* promotes CXCL8 release from intestinal epithelial cells

**Figure 1.**
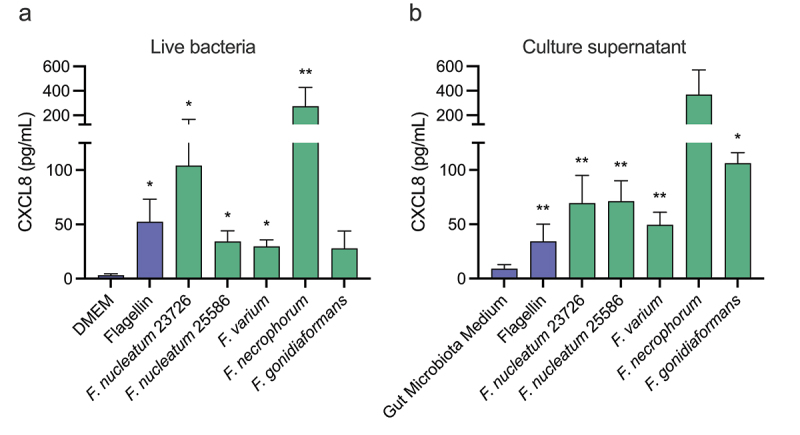
*Fusobacterium* species induce CXCL8 secretion from intestinal epithelial cells. HT-29 intestinal epithelial cells were stimulated with (a) living *Fusobacterium* species or (b) sterile conditioned culture medium from *Fusobacterium* strains for 24 h, after which the CXCL8 secretion was measured. As controls, bacterial medium, cell culture medium (DMEM) or purified flagellin was used. Values represent the mean ± SEM of three independent experiments performed in duplicate. Ratio paired t tests comparing stimulations to DMEM or GMM were used for statistical analysis. *p < 0.05, **p < 0.01.

Chronic inflammation is a key driver of tumorigenesis, for example through elevated CXCL8 levels.^20–22^ As *Fusobacterium nucleatum* has been shown to continuously reside on and in tumor cells, we examined whether the proximity of *F. nucleatum* could promote the induction of pro-inflammatory responses in these cells. For this, we infected HT-29 intestinal tumor epithelial cells for 24 h with two different *F. nucleatum* subsp. nucleatum strains (ATCC 25,586 and ATCC 23,726) and measured CXCL8 release by ELISA. As shown in Figure 1a, both *F. nucleatum* strains significantly promoted CXCL8 release. To examine whether this effect was specific for *F. nucleatum* or conserved among other *Fusobacterium* species, HT-29 cells were subsequently infected with living clinical isolates of *Fusobacterium varium*, *Fusobacterium periodonticum* and *Fusobacterium necrophorum*. While there was some variation in the CXCL8 release between individual experiments, all *Fusobacterium* spp. showed a clear trend toward the induction of CXCL8 release in HT-29 cells (Figure 1a). While *Fusobacterium* spp. are known to adhere to epithelial cells, the adhesins involved in binding are not conserved within *Fusobacterium* spp. Therefore, we hypothesized that CXCL8 induction is not dependent on binding to the epithelium, but caused by a released compound. So, we isolated cell-free bacterial conditioned culture medium from the set of *Fusobacterium* strains and stimulated HT-29 cells. Similar to what was observed with living intact bacteria, all *Fusobacterium* culture media were able to induce CXCL8 release, suggesting that compounds released from various *Fusobacterium* spp. possess pro-inflammatory activity (Figure 1b).

### Chemical characterization identifies the proinflammatory *Fusobacterium* factor as a small putative carbohydrate

**Figure 2.**
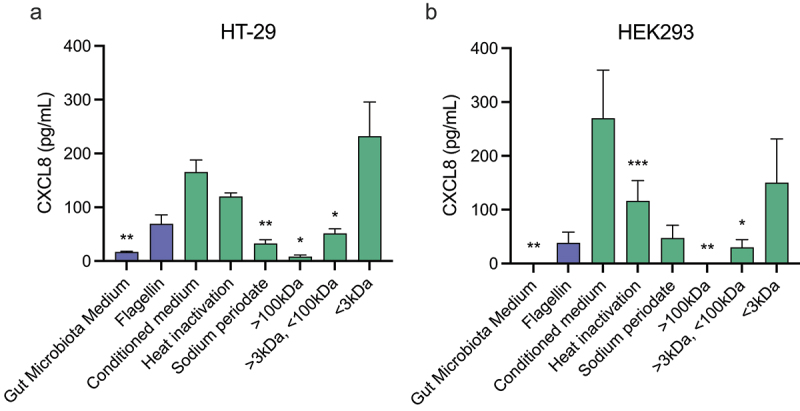
The *Fusobacterium*-released pro-inflammatory factor is a small carbohydrate. Cell-free bacterial conditioned culture medium from F. nucleatum 23,726 was subjected to heat inactivation, sodium periodate treatment and size-based fractionation. (a) HT-29 cells were stimulated with the F. nucleatum 23,726 culture medium or the various treated culture media and CXCL8 release after 24 h of stimulation was measured (b) HEK293T cells were treated similarly as in a). Values represent mean ± SEM of three independent experiments performed in duplicate. Ratio paired t test comparing stimulations to conditioned medium were used for statistical analysis. *p < 0.05, **p < 0.01, ***p < 0.001.

To investigate what type of molecule from the *Fusobacterium*-conditioned medium induces CXCL8 secretion, we performed several treatments of the conditioned medium to narrow down the responsible factor. First, the cell-free conditioned culture medium of *F. nucleatum* 23726 was heat-inactivated to denature potential pro-inflammatory proteins present in the conditioned medium (Figure 2a). Heat-inactivated conditioned medium resulted in a similar CXCL8 release from HT-29 cells as the untreated conditioned medium, suggesting that the inflammatory compound is heat-resistant and thus likely not of proteinaceous nature. Next, the conditioned medium was treated with sodium periodate, which oxidizes carbohydrates. Sodium periodate treatment almost completely abrogated the pro-inflammatory effect of *F. nucleatum* 23726 conditioned medium, indicating that a carbohydrate was involved in the *F. nucleatum*-induced immune activation. Pre-treatment of HT-29 cells with sodium periodate did not led to decreased CXCL8 release after stimulation with cell-free conditioned culture medium of *F. nucleatum* 23726, indicating that the treatment did not affect a cellular receptor or signaling pathway (Figure S1). Finally, we fractionated the conditioned medium based on molecular weight to reveal the size of the pro-inflammatory compound. The fraction containing only molecules smaller than 3 kDa induced similar CXCL8 release from HT-29 cells as the untreated conditioned medium, while the fraction containing molecules between 3 kDa and 100 kDa showed a significant reduction in CXCL8 release. The fraction containing molecules larger than 100 kDa lost all CXCL8-inducing activity. Thus, these results suggest that *F. nucleatum*-induced CXCL8 release in HT-29 cells is caused by a small, heat-stable putative carbohydrate(−containing) molecule.

### *Fusobacterium* promotes NF-κB activation in an ALPK1-dependent manner in HEK293 cells

**Figure 3.**
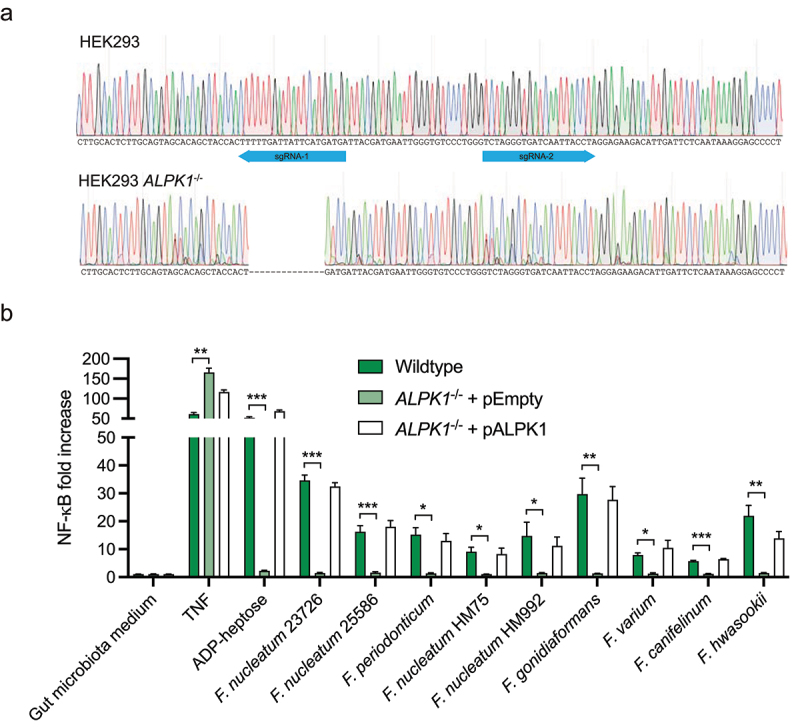
*Fusobacterium* species induce NF-κB activation in an ALPK1-dependent manner in HEK293 cells. a) the genetic mutation in the ALPK1 gene in CRISPR-Cas9 edited HEK293 ALPK1^−/−^ cells was validated using sanger sequencing. b) HEK293 wildtype cells, HEK293 ALPK1^−/−^ cells supplemented with an empty vector control or ALPK1^−/−^ cells transfected with an ALPK1 complementation plasmid were stimulated with TNF, adp-heptose, bacterial culture medium or bacterial conditioned culture medium of various *Fusobacterium* species. NF-κB activation was measured after 5.5 h stimulation and calculated as fold increase over the bacterial medium control. Values represent mean ± SEM of three independent experiments performed in triplicate. Unpaired t tests were used for statistical analysis of HEK293 cells compared to HEK293 ALPK1^−/−^ cells. *p < 0.05, **p < 0.01, ***p < 0.001.

The characteristics of the *F. nucleatum*-released molecule that induced CXCL8 release are similar to those of ADP-heptose, a previously identified bacterial metabolite that activates the intracellular immune receptor ALPK1.^23^ Alternatively, it could indicate a related heptose phosphate, such as heptose-1,7-bisphosphate.^24^ To test the hypothesis that ADP-heptose and/or a related heptose phosphate is the *F. nucleatum*-released molecule inducing CXCL8 release, we first stimulated the ALPK1-expressing HEK293 reporter cell line with heat-treated, sodium periodate treated and size-fractionated *F. nucleatum* 23726 conditioned medium (Figure 2b). Due to the limited number of pattern recognition receptors expressed, HEK293 cells form a simplified model system that allows the investigation of ADP-heptose-mediated NF-κB responses more specifically that in HT-29 cells. Similar to what we observed using the HT-29 cells the release of CXCL8 in HEK293 cells was dependent on a small, heat-stable, putative carbohydrate(−containing) molecule. The release of CXCL8 following activation of ALPK1 has been shown to occur in an NF-κB-dependent manner.^25^ To investigate whether *Fusobacterium* could activate ALPK1 and NF-κB, we generated a HEK293 *ALPK1*^−/−^ cell line using CRISPR-Cas9 gene editing and transfected the cells with an NF-κB luciferase reporter (Figure 3a). After stimulation of wild-type HEK293 cells with the conditioned medium of an array of *Fusobacterium* species, including- *F. nucleatum*, *F. periodonticum*, *F. gonidiaformans*, *F. varium*, *F. canifelinum* and *F. hwasookii*, we observed potent NF-κB activation for all strains (Figure 3b). In contrast, stimulation of HEK293 *ALPK1*^−/−^ cells with conditioned culture medium from the same *Fusobacterium* species showed a complete lack of NF-κB activation, suggesting a key role for ALPK1 in *Fusobacterium*-induced pro-inflammatory responses. To confirm that the deletion was restricted to ALPK1 and not the result of off-target mutations that were introduced during CRISPR-Cas9-mediated gene editing, the HEK293 *ALPK1*^−/−^ cells were complemented with a plasmid that constitutively expressed ALPK1. Following stimulation of the complemented HEK293 *ALPK1*^−/−^ cells, all responses were restored to comparable levels of the wild-type cells, confirming that the observed NF-κB activation was fully ALPK1-dependent. Finally, we examined the specificity of this phenotype for *Fusobacterium* species by testing three other intestinal bacterial isolates for the presence of ALPK1-activating secreted compound. While purified ADP-heptose and *Fusobacterium* supernatant showed a significant activation of NF-κB, none of the other bacterial isolates showed this effect (Figure S2). Based on these results, we conclude that *Fusobacterium* species secrete ADP-heptose or related heptose phosphates to promote an inflammatory response through ALPK1.

### *Fusobacterium* promotes CXCL8 release in an ALPK1-dependent manner in HT-29 cells

**Figure 4.**
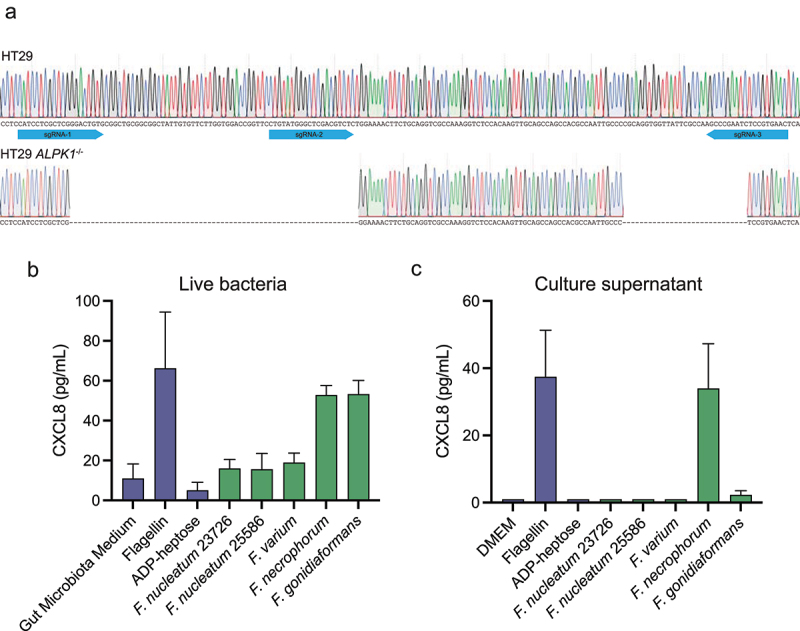
*Fusobacterium*-conditioned culture medium and live co-culture induce limited CXCL8 in HT-29 ALPK1^−/−^ cells. (a) the genetic mutation in the ALPK1 gene in CRISPR-Cas9 edited HT-29 ALPK1^−/−^ cells was validated using sanger sequencing. (b) CXCL8 concentrations of HT-29 ALPK1^−/−^ cells stimulated with bacterial culture medium or indicated controls for 24 h. c) CXCL8 concentrations of HT-29 ALPK1^−/−^ cells stimulated with living bacterial cultures at an MOI of 100 or indicated controls for 24 h. Values represent mean ± SEM of two or three independent experiments performed in triplicate. Ratio paired t-test comparing stimulations to GMM or DMEM were used for statistical analysis.

As our results showed that *Fusobacterium* is able to activate NF-κB in an ALPK1-dependent manner in HEK293 cells, we next set out to assess the role of ALPK1 in *Fusobacterium*-induced CXCL8 release in intestinal HT-29 cells. Therefore, we employed CRISPR-Cas9 gene editing to create an *ALPK1*^−/−^ knockout in HT-29 cells. Sequencing analysis confirmed that ALPK1 was effectively disrupted (Figure 4a). Stimulation of HT-29 *ALPK1*^−/−^ cells with ADP-heptose showed absence of CXCL8 release, clearly showing the functional effect of the *ALPK1* deletion (Figure 4b,c). Stimulation of the cells with sterile conditioned medium of *F. nucleatum* and *F. varium*, showed a complete loss of CXCL8 release in HT-29 *ALPK1*^−/−^ cells (Figure 4b) relative to the CXCL8 release in wildtype HT-29 cells (Figure 1b). This suggests that conditioned medium of these strains activated HT-29 cells via ALPK1 (Figure 4b). *F. necrophorum* and *F. gonidiaformans* conditioned medium still promoted CXCL8 release from HT-29 *ALPK1*^−/−^ cells, albeit at lower levels than observed in wildtype HT-29 cells (Figure 1b) suggesting that these bacteria also produce additional pro-inflammatory molecules. Co-culturing with living *Fusobacterium* strains indicated a similar loss of CXCL8 induction in HT-29 *ALPK1*^−/−^ cells compared to wildtype HT-29, except for *F. necrophorum* (Figures 1a and 4c). Since *Fusobacterium* species thrive under low oxygen conditions, we finally set out to examine whether co-culturing of HT-29 cells with live *F. nucleatum* under hypoxic, 5% O_2_ conditions significantly affected the cellular response to the bacteria. As shown in Figure S3, HT-29 cell grown under 5% O_2_ responded equally well to ADP-heptose, suggesting that ALPK1 signaling is not influenced by hypoxia. In contrast, the response to live *F. nucleatum* was significantly higher when co-culturing occurred at 5% O_2_, showing either that increased growth or survival of *F. nucleatum* result in more ADP-heptose release, or that *F. nucleatum* produced more ADP-heptose under these conditions. Altogether, our data indicate that the secreted molecule by multiple *Fusobacterium* species likely is ADP-heptose and/or a related heptose phosphate, which signals through ALPK1 in both HT-29 and HEK293 cells to promote the release of CXCL8 and/or activation of NF-κB.

### ADP-heptose regulates the expression of inflammation and cancer-related pathways

**Figure 5.**
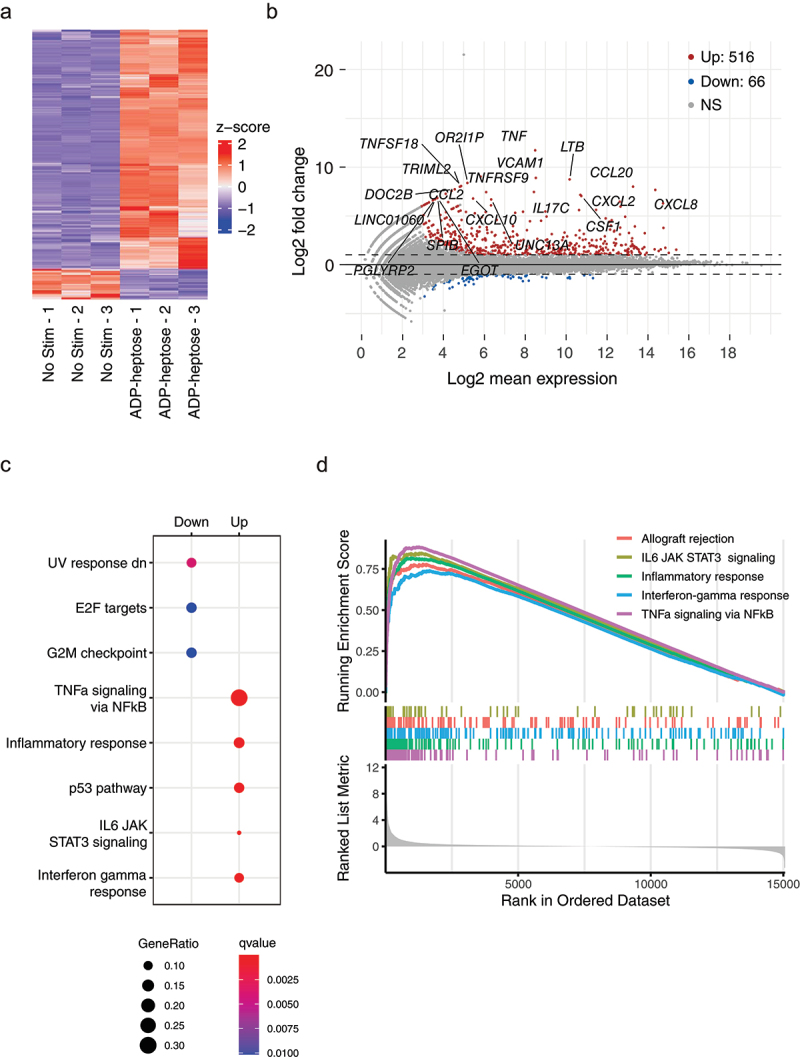
Adp-heptose promotes a largely pro-inflammatory and pro-carcinogenic signature in HT-29 cells. a) Heatmap of the significantly up- and downregulated genes in HT-29 cells stimulated with adp-heptose compared to non-stimulated (no stim) cells. b) volcano plot of the up- and downregulated genes resulting from adp-heptose stimulation, a number of highly upregulated genes is indicated. c) MSigDB Hallmark over-representation gene set analysis of top five up- and downregulated sets of genes. d) gene set enrichment analysis (GSEA) plots using MSigDB gene sets. The top half shows the running enrichment score for the top five most differentially regulated gene sets (indicated by different colors), colored bars indicate the position of each of the members of the gene sets along the ranked gene list (rank in ordered dataset). Bottom half shows the ranked list metric as bar plot (log2 fold change), which measures the degree of correlation of genes with the gene sets (left for positive and right for negative correlation).

**Figure 6.**
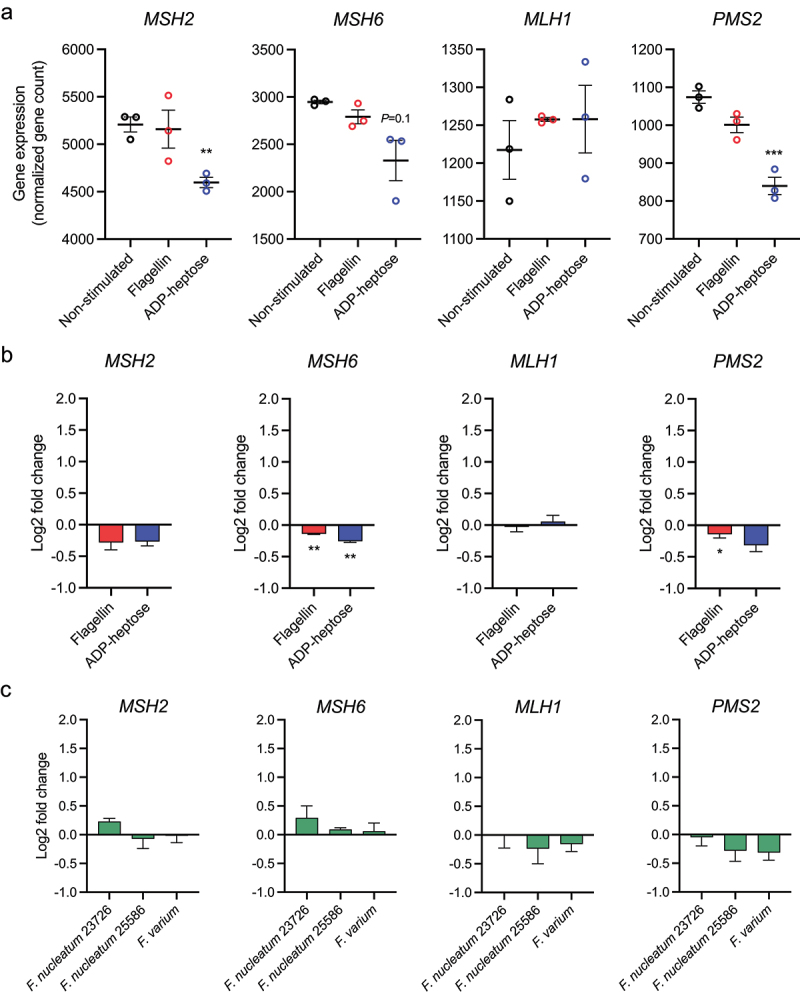
The impact of adp-heptose stimulation on DNA mismatch repair gene expression. a) gene expression in fragments per kilobase of transcript per million mapped reads (FPKM) of MSH2, MSH6, MLH1 and PMS2 from RNA-sequencing results of non-stimulated, flagellin-stimulated or adp-heptose-stimulated HT-29 cells from three independent experiments. Statistical testing under these three conditions was performed using the empirical Bayesian hierarchical modeling approach encoded in R package EBSeq. ** adjusted *p* value < 0.01, *** adjusted *p* value < 0.001. b) HT-29 cells were stimulated with adp-heptose, flagellin or non-stimulated (NS) for 2.5 h and expression of indicated genes was assessed by rt-qPCR. Values are indicated as log2 fold change over the non-stimulated control. Statistical testing was performed by comparing adp-heptose or flagellin to control stimulation. c) HT-29 cells were stimulated with medium control or bacterial conditioned culture medium of indicated strains for 2.5 h and expression of indicated genes was assessed by rt-qPCR. Values are indicated as log2 fold change over the non-stimulated control. Statistical testing was performed by comparing bacterial strains to medium control stimulation. For b and c, values represent mean ± SEM of three independent experiments performed in triplicate and an paired t test was used for statistical analysis. *p < 0.05, **p < 0.01, ***p < 0.001.

To subsequently understand the effects that *Fusobacterium* has on epithelial (tumor) cells, we assessed global RNA transcription in HT-29 cells that were stimulated with ADP-heptose using RNA-sequencing. ADP-heptose stimulation resulted in the statistically significant upregulation of 516 genes, while 66 genes were downregulated (Figure 5a,b and Table S2). As observed in Figure 5b, the upregulated genes had, on average, a higher fold-difference in expression and a higher significance. Among the top upregulated genes, the majority are key pro-inflammatory genes, such as *TNF, CCL20* and *CXCL8* (Figure 5b), while the downregulated genes contained various DNA binding proteins, lncRNAs and pseudogenes. When investigating differentially expressed MSigDB Hallmark pathways, most enriched pathways were inflammation-related, such as pathways related to “TNFα signaling via NF-κB” or “Interferon gamma response” (Figure 5c). Similarly, the gene sets with the highest running enrichment scores were the same, with “Allograft rejection” as additional gene set (Figure 5d). To investigate the transcriptional similarities and differences between ADP-heptose/ALPK1-mediated cellular activation and a hallmark innate immune NF-κB-inducing PAMP, the transcriptional response to bacterial flagellin in HT-29 cells was also determined. Fewer genes were differentially regulated (43 up, 2 down) in response to flagellin, while most of the inflammation-related MSigDB Hallmark pathways were overlapping between ADP-heptose- and flagellin-stimulated cells, such as “TNFα signaling via NF-κB” and “Interferon gamma response” (Figure S3A-C and Table S3). Additionally, the gene sets with the highest running enrichment scores of the flagellin-stimulated cells overlapped exactly with ADP-heptose (Figure S3D). Considering the role of *Fusobacterium* in cancer development, we next examined which cancer-related genes MSigDB Hallmark pathways were differentially regulated as result of ALPK1 activation (Figure 5c). Upregulated pathways included “IL6 JAK STAT3 signaling” and “P53 pathway”, while the downregulated pathways were related to cell-cycle control and DNA damage response, namely, “UV response down (dn)”, “E2F targets” and “G2M checkpoint”. All upregulated pathways in response to ADP-heptose can also be observed in the flagellin stimulated cells, except for P53 pathway (Figure S3C and D). In contrast, the downregulated pathways as result of ADP-heptose stimulation were not observed in flagellin stimulated cells, indicating it is a unique result of ADP-heptose stimulation. As the downregulated pathways largely relate to consequences of DNA damage, and *Fusobacterium* prevalence has been widely associated with CRC with defective DNA mismatch repair, we examined the four genes that are essential for DNA mismatch repair: *MSH2*, *MSH6*, *PMS2* and *MLH1*. ADP-heptose stimulation resulted in a moderate but significant downregulation of two out of these four genes, while only one of these genes was downregulated by flagellin (Figure 6a). Therefore, RT-qPCR was performed to confirm the downregulation of the DNA mismatch repair genes compared to flagellin stimulation. Only *MSH6* showed significant downregulation after ADP-heptose stimulation, while *MSH2* and *PMS2* were not significantly downregulated compared to flagellin-stimulated HT-29 cells (Figure 6b). In addition, *F. nucleatum*-conditioned medium did not result in downregulation of DNA mismatch repair genes, perhaps highlighting the subtlety of the phenotype (Figure 6b,c). Finally, we investigated whether the effect of ADP-heptose were specific for tumor cells or could also be observed in healthy intestinal epithelial cells. For this purpose, healthy rectal organoids were stimulated with ADP-heptose or flagellin and RNA expression was analyzed by RNA-SEQ. While the healthy organoids responded to flagellin by initiating a pro-inflammatory gene program similar to what was observed in HT-29 intestinal tumor cells (Figure S4a and b), ADP-heptose failed to induce alterations to gene expression, suggesting that the ALPK1/TIFA signaling pathway is not functionally expressed in these cells (Figure S4c).

### ADP-heptose-induced ALPK1 activation increases the NF-κB-dependent expression of the checkpoint inhibitor protein PD-L1

A deficiency of the DNA mismatch repair machinery resulting in microsatellite instability (MSI)- positive tumors is characterized by a high mutational burden and is also associated with an upregulation of the immune checkpoint inhibitory receptor PD-L1 and an increased sensitivity to anti-PD-(L)1 immunotherapy.^26,27^ Additionally, *Fusobacterium nucleatum* has been previously shown to induce the expression of PD-L1 in tumor cells, while also increasing the sensitivity to anti-PD-L1 immunotherapy, but the molecular mechanism behind the upregulated PD-L1 by *Fusobacterium* remains unresolved.^11,12^ We therefore examined our transcriptional dataset to investigate whether ADP-heptose stimulation affected *PD-L1* transcription. As shown in Figure 7a, ADP-heptose significantly increased the expression of *PD-L1* in HT-29 cells, while flagellin did not. These results were confirmed by RT-qPCR, where ADP-heptose induced a significant upregulation of *PD-L1* compared to flagellin (Figure 7b). Importantly, we confirmed that the upregulation of *PD-L1* is indeed dependent on ALPK1 as shown by the absence of ADP-heptose-dependent *PD-L1* upregulation in HT-29 *ALPK1*^−/−^ cells. Both *F. nucleatum* and *F. varium*-conditioned medium also promoted moderate but significant upregulation of *PD-L1* compared to the medium control (Figure 7c). This effect was also confirmed to be dependent on ALPK1, since HT-29 *ALPK1*^−/−^ cells did not show differential *PD-L1* expression. Also, the upregulation of *PD-L1* by ADP-heptose was show to be time and dose-dependent (Figure 7d,e). While PD-L1 mostly affects the interaction between tumor cells and T cells, increasing evidence suggests that intrinsic PD-L1 signaling may impact the proliferation and survival of tumor cells.^28^ However, within our experimental setup, stimulation of HT-29 cells with ADP-heptose, while increase *PD-L1* expression, did not affect the proliferation of the cells (Figure S6).

**Figure 7.**
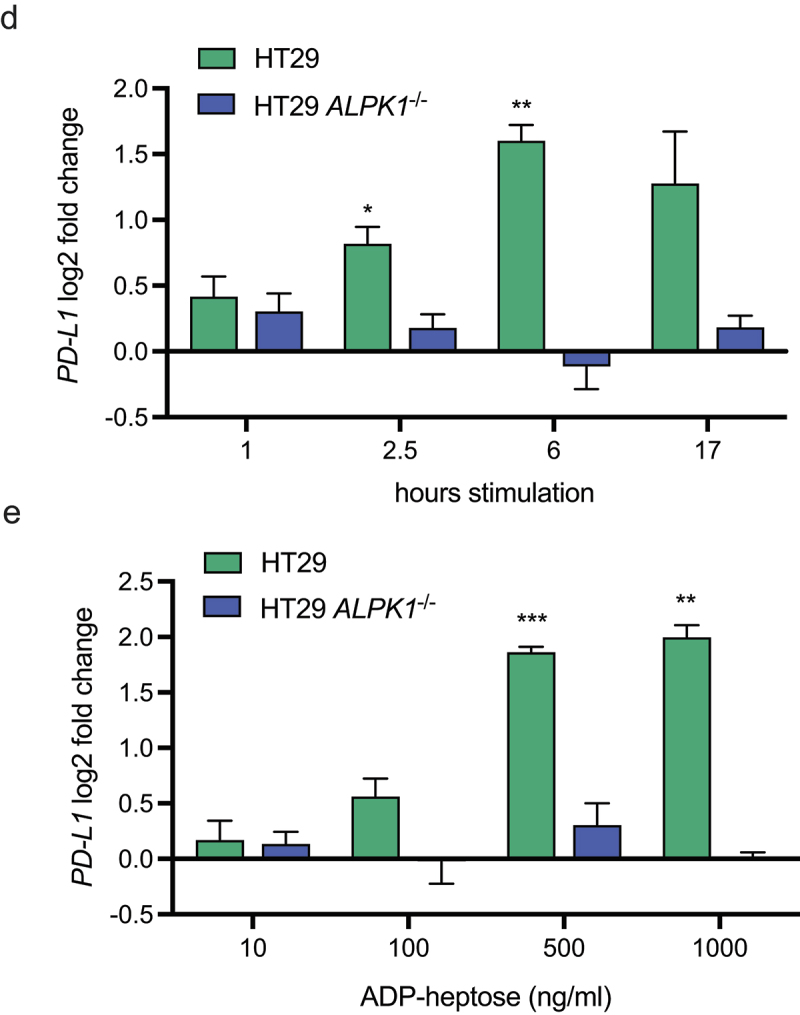
(Continued).

**Figure 7.**
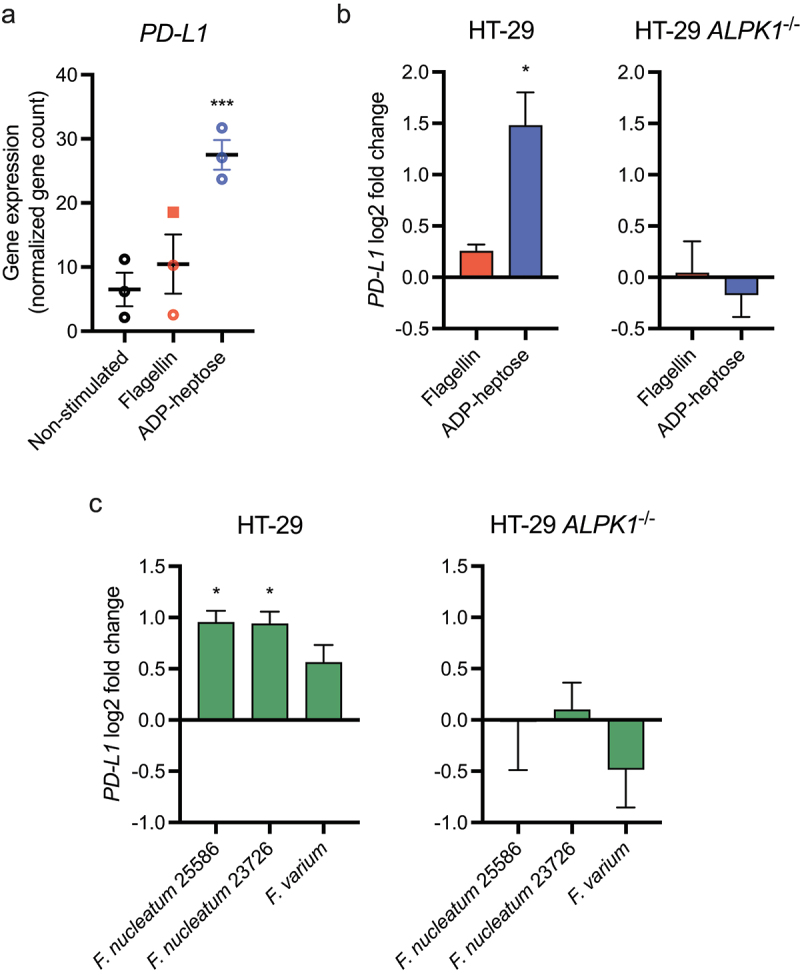
Adp-heptose and *F. nucleatum* upregulate *PD-L1* in HT-29 cells in an ALPK1-dependent manner. (a) gene expression in fragments per kilobase of transcript per million mapped reads (FPKM) of *PD-L1* from RNA-sequencing results of non-stimulated, flagellin-stimulated or adp-heptose-stimulated HT-29 cells from three independent experiments. Statistical testing under these three conditions was performed using the empirical Bayesian hierarchical modeling approach encoded in R package EBSeq. *** adjusted *p* value < 0.001. (b) HT-29 cells or HT-29 *ALPK1*^−/−^ cells were stimulated with adp-heptose, flagellin or non-stimulated (NS) for 2.5 h and expression of *PD-L1* was assessed by rt-qPCR. Values are indicated as log2 fold change over the non-stimulated control. Statistical testing was performed by comparing adp-heptose or flagellin to control stimulation. (c) HT-29 cells or HT-29 *ALPK1*^−/−^ cells were stimulated with bacterial conditioned culture medium from indicated strains or medium control. Values are indicated as log2 fold change over the non-stimulated control, statistical analysis was performed by comparing *Fusobacterium* strains to the medium control. (d) *PD-L1* expression in HT-29 cells or HT-29 *ALPK1*^−/−^ cells stimulated with ADP-L-heptose (500 ng/ml) for the indicated amount of time. Values are indicated as log2 fold change over the non-stimulated control, was performed by comparing adp-heptose conditions to control stimulation. E) *PD-L1* expression in HT-29 cells or HT-29 *ALPK1*^−/−^ cells stimulated for 6 h with indicated amounts of ADP-L-heptose. Values are indicated as log2 fold change over the non-stimulated control, statistical analysis was performed by comparing adp-heptose conditions to control stimulation. For B-E, values represent mean ± SEM of three independent experiments performed in triplicate. A paired t test was used for statistical analysis. *p < 0.05, **p < 0.01, ***p < 0.001.

**Figure 8.**
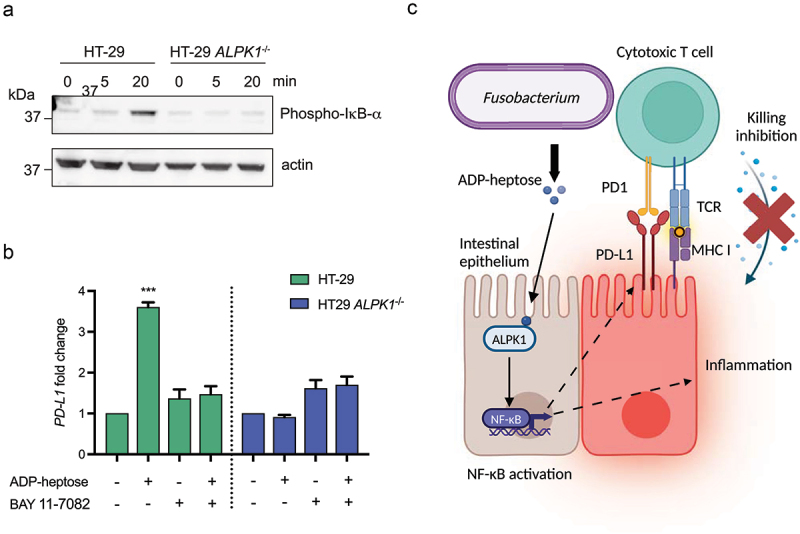
Adp-heptose-mediated upregulation of *PD-L1* is dependent on NF-κB. a) adp-heptose stimulation results in the phosphorylation of IκBα in HT-29 as determined by western blot analysis. b) inhibition of NF-κB by BAY 11–7082 abrogates adp-heptose-mediated upregulation of *PD-L1* expression. Values are indicated as fold change over the non-stimulated control, statistical analysis was performed by comparing adp-heptose conditions to control stimulation. An unpaired t test was used for statistical analysis. ***p < 0.001. c) schematic overview of the effects of *fusobacterium*-derived adp-heptose in colorectal cancer cells.

PD-L1 has been shown by various studies to be regulated by the transcription factor NF-κB.^29,30^ In order to elucidate the mechanism behind the ADP-heptose-dependent upregulation of *PD-L1*, we therefore examined the activation of NF-κB in HT-29 following ADP-heptose stimulation by monitoring the phosphorylation of IκBα. Phosphorylation of IκBα liberated NF-κB and allows for its transfer to the nucleus, where it induced transcriptional changes. Using an antibody specific for phosphorylated IκBα, we revealed that IκBα became phosphorylated at 20 min post-stimulation with ADP-heptose (Figure 8a) Stimulation of HT-29 ALPK1^−/−^ cells did not show changes in the phosphorylation status of IκBα, showing that the effect was dependent of an activated ALPK1 pathway. Inhibition of NF-κB activation by the selective inhibitor BAY 11–7082 resulted in a complete abrogation of ADP-heptose-mediated PD-L1 upregulation, which again was ALPK1-dependent (Figure 8b). Combined, these results show that ADP-heptose-mediated ALPK1-dependent upregulation of *PD-L1* by *Fusobacterium* is dependent on the activation of transcription factor NF-κB.

## Discussion

The manner in which tumor-associated *Fusobacterium nucleatum* contributes to a pro-inflammatory tumorigenic environment remains elusive. In the current study, we reveal how the LPS biosynthesis metabolite ADP-heptose, released by *F. nucleatum*, contributes to a pro-inflammatory and pro-tumorigenic environment in an ALPK1-dependent manner (Figure 8c). We uncover that not only *Fusobacterium nucleatum* subsp. nucleatum but also other (sub)species of *Fusobacterium* secrete ADP-heptose or related heptose phosphates, resulting in ALPK1-dependent activation of NF-κB and release of CXCL8. The fact that most other members of the *Fusobacterium* genus that were examined exhibited a similar phenotype shows strong evolutionary conservation of this immune-activating mechanism. Through RNA-sequencing, we elucidate that ADP-heptose- stimulated ALPK1 also alters several non-inflammatory pathways, such as pathways involved in response to DNA damage and the p53 pathway, which are both be linked to colorectal cancer. Lastly, we find that ADP-heptose and *Fusobacterium nucleatum* subsp. nucleatum promote NF-κB-dependent expression of the inhibitory receptor *PD-L1*.

Various lines of evidence exist on the ability of *Fusobacterium* to induce the release of CXCL8 in different cell lines and types by either bacterial conditioned medium or infection with living bacterial cells.^21,31–33^ Most studies have been performed using the HCT116 colorectal cancer cell line, which releases CXCL8 after stimulation with living *F. nucleatum*.^21,32^ Also, HT-29 cells were shown to respond to co-culture with *F. nucleatum* by upregulating CXCL8 and other pro-inflammatory genes.^34^ This is in accordance with our own research, which identifies all *Fusobacterium* strains tested to be inducers of CXCL8 in HT-29 cells. The factor(s) responsible for the immune activation appears to be different in the various studies compared to our own findings. For instance, it was found that HT-29 cells solely responded to the > 50 kDa fraction of *F. nucleatum*-conditioned medium and purified *F. nucleatum* outer membrane vesicles (OMVs).^31^ In contrast, we find in the present study that the CXCL8-inducing factor is highly enriched in the < 3 kDa fraction of *Fusobacterium-*conditioned medium, while bigger fractions have significantly lower CXCL8-inducing capabilities. While this suggests the possibility of multiple different modes of innate immune stimulation by released factors from *Fusobacterium*, we find that, with our experimental setup, ALPK1 is the sole receptor leading to the CXCL8 release in HT-29 cells for *F. nucleatum* and *F. varium*, but not *F. necrophorum* and *F. gonidiafromans*. This suggests that only ALPK1-activating ligands are released by *F. nucleatum* under our experimental conditions. It is important to note that different bacterial culture media were used to cultivate *Fusobacterium*, which could have a significant effect on released metabolites. In addition, different strains of *Fusobacterium* might release different activators of CXCL8. We also show that co-culturing of *F. nucleatum* with HT-29 cells under 5% O_2_ conditions results in a modest but significant increase in CXCL8 release. While we did not provide definitive evidence that this increase is ALPK1-dependent, it is likely that the lower oxygen levels resulted in increased bacterial growth and thereby more ADP-heptose release. This is supported by a study examining the role of hypoxia during *F. nucleatum* infections, which finds that low oxygen is a crucial factor in the outcome of tumorigenesis.^35^ *F. nucleatum-*conditioned medium has also been reported to induce CXCL8 secretion in human gingival fibroblasts.^32^ It would be interesting to determine if this response is also ALPK1-dependent and whether the ADP-heptose-ALPK1-NF-κB axis would also function in other (cancer-associated) fibroblasts, as inflammation in these cells has been reported to play an essential role in skin tumorigenesis.^36^ To support this potential research angle, presence of *Fusobacteriales* has been particularly associated with a worse outcome in patients with a mesenchymal phenotype of colorectal cancer.^37^

We find that *Fusobacterium* promotes NF-κB activation and CXCL8 release in an ALPK1-dependent manner. Recently, a different study showed that co-cultures with live *F. nucleatum* induced an NF-κB response that was abrogated upon knockdown of *ALPK1* in the previously mentioned HCT116 cell line.^38^ Afterwards, another study showed that *F. nucleatum* conditioned culture medium promotes NF-κB activation.^39^ By applying CRISPR-Cas9-mediated gene inactivation in two separate cell lines (HT-29 and HEK293) we come to the conclusion that not only *F. nucleatum* subsp. nucleatum but also various other *Fusobacterium* species are able to induce an NF-κB response and CXCL8 release that is indeed dependent on ALPK1. The previously mentioned studies identifies ALPK1-dependent upregulation of *ICAM1* as a possible contributor to *Fusobacterium*-induced metastasis of colorectal cancer and that *F. nucleatum*, but not ADP-heptose, promoted cell proliferation and anti-apoptotic effects.^38,39^ In our study, we looked into the effects of ADP-heptose on global gene expression, and find that ADP-heptose upregulates a wide range of genes, many of which indeed could play a role in development and progression of colorectal cancer.^38^ Notably, the responsible compound for ALPK1 activation in the former study was not further delineated. Here, we provide further evidence that the responsible compounds is ADP-heptose and/or related heptose by biochemical characterization. To further conclusively determine that ADP-heptose and not another heptose phosphate or (novel) metabolite is responsible for the *Fusobacterium*-induced ALPK1 activation, genetic modification of the ADP-heptose synthesis pathway in *Fusobacterium* would be required. However, attempts at genetically inactivating the ADP-heptose synthesis gene *HldA* in *F. nucleatum* have been unsuccessful thus far. This may indicate that the ADP-heptose synthesis pathway is essential in *F. nucleatum*. On the other hand, this seems unlikely given that the ADP-heptose synthesis pathway can be modified in several other bacteria, for example *C. jejuni* (*HldE*) and *H. pylori* (*RfaE*).^15,40^

We found that, among other pathways, the DNA mismatch repair system that includes the genes *MSH2*, *MSH6*, *MLH1* and *PMS2*^41^ was downregulated upon ADP-heptose stimulation. DNA mismatch repair is essential in the repair of nucleotide mismatches that primarily occur after DNA replication. A dysfunctional or downregulated DNA mismatch repair system can result in accumulation of DNA insertions and deletions that ultimately lead to a high mutational burden.^41^ One or more of the genes involved in DNA mismatch repair are often found to be mutated in colorectal cancer, which results in microsatellite instability (MSI), characterized by a high mutational burden.^41^ We found that ADP-heptose downregulated two out of these four genes, *MSH2* and *PMS2*. Interestingly, the presence of *F. nucleatum* has been found to be more strongly associated with MSI-high tumors compared to microsatellite stable (MSS) tumors.^42^ Other research has subsequently confirmed this association, demonstrating that *F. nucleatum* not only occurs more frequently, but also more abundantly in MSI-high tumors.^34,43,44^ Additionally, in both patients with the non-hereditary MSI-high variants as in patients with the hereditary MSI-high variant, called Lynch syndrome, significantly more *F. nucleatum* were detected than in microsatellite stable patients.^44^ Our data therefore indicates that *F. nucleatum* may directly contribute to the MSI phenotype in tumors via the release of ADP-heptose, the activation of ALPK1 and subsequent downregulation of the DNA mismatch repair pathway in the epithelium or tumor cells. While the potential mechanism of downregulation by ALPK1 signaling remains elusive, it could be hypothesized to involve alterations of methylation patterns or DNA mutations in the promoters of the various mismatch repair genes. However, it must be stated that the effects on this pathway are minimal and only clearly observed using RNA-SEQ analysis. To further strengthen our results, functional experiments would be required to show a direct effect of *F. nucleatum* released ADP-heptose on DNA mismatch repair and the accumulation of mutations, for instance by using mismatch repair reporter cell systems.^45^

A result of microsatellite instability is the increased number of produced neo-antigens compared to microsatellite stable tumors, which makes tumors more effectively recognized by immune cells (cytotoxic T cells). Additionally, MSI-high colorectal cancer is more sensitive to immune checkpoint inhibitor (ICI) therapy, while MSS tumors largely remain unresponsive.^46^ The reasons behind this selective responsiveness are believed to be associated with the high mutational burden and heightened levels of tumor-infiltrating lymphocytes inherent to CRC MSI-high tumors.^46^ In the current study, we find that, in addition to the downregulation of DNA mismatch repair genes, *PD-L1* (*CD274*) is upregulated, which indicates that ADP-heptose could promote an immune-suppressive tumor microenvironment. As effective evasion of recognition and killing by the immune system is an essential aspect of tumorigenesis, *F. nucleatum*-mediated ALPK1 activation and subsequent PD-L1 upregulation may play an important role in pathogenesis. Conversely, it could also indicate that tumors in which ALPK1 is being activated by ADP-heptose-releasing *F. nucleatum* are more responsive to ICI therapy. Indeed, it has been reported that presence of *F. nucleatum* in CRC correlates with an increased response to ICI therapy, which is suggested to be promoted in an NF-κB and PD-L1-dependent manner.^12^ Whether *F. nucleatum* causes an immune-suppressive tumor environment in an ADP-heptose dependent manner and whether this has consequences for ICI therapy remains to be further explored using *in vivo* models. This essential next step should be taken to directly link the ADP-heptose-ALPK1-TIFA axis to both a proinflammatory tumor microenvironment and an upregulated PD-L1. These studies could be conducted in mouse tumor models with ADP-heptose-deficient *F. nucleatum* but, as already mentioned above, are currently limited by the lack of an ADP-heptose-deficient *Fusobacterium* strain, Alternatively, ALPK1/TIFA-deficient mice could be used. We further demonstrate that healthy intestinal epithelial cells, under homeostatic conditions, appear unable to respond to ADP-heptose. While this might suggest that *Fusobacterium*-derived ADP-heptose mainly exert its effect at a later stage of tumorigenesis, more studies are needed to assess whether the unresponsiveness to ADP-heptose is observed throughout the entire healthy intestinal tract.

Because of the association of *F. nucleatum* with colorectal cancer, we have focused on the perspective of the intestinal tract and colorectal cancer. However, in addition to being a colonizer of the gastrointestinal microbiota, *F. nucleatum* is particularly known as colonizer in the human oral microbiota. Additionally, *Fusobacterium* can be involved in multiple pathologies throughout the human body.^47^ The most prominent example is Lemierre’s syndrome, a bacteremia with thrombophlebitis of the internal jugular vein, which is most often caused by *Fusobacterium necrophorum*.^48–50^ Additionally, *Fusobacterium nucleatum* has been associated with multiple diseases, including periodontitis, inflammatory bowel disease and adverse pregnancy outcomes.^47^ In relation to periodontitis, *F. nucleatum* can promote an inflammatory response in both the oral epithelium, fibroblasts and immune cells.^47^ In all these *Fusobacterium*-associated disorders, however, the exact role of *Fusobacterium* in the pathogenesis of disease and the molecular mechanisms behind the diseases remain largely unknown. Since we have found that all *Fusobacterium* species tested secrete the pro-inflammatory ADP-heptose or related heptose phosphates, it would be of interest to further explore the role of ADP-heptose in these pathologies.

In conclusion, we show that various species of the *Fusobacterium* genus are able to activate ALPK1 in colorectal cancer cells via the release of ADP-heptose or other ALPK1-activating heptose phosphates into its environment. The consequence of ADP-heptose-mediated ALPK1 activation in these cells is a general pro-inflammatory response (which can be tumor-promoting), the downregulation of DNA mismatch repair genes and the upregulation of the checkpoint inhibitor protein PD-L1. Our findings contribute to the elucidation of the interaction between *F. nucleatum* and intestinal epithelial tumor cells, may explain why *Fusobacterium*-associated tumors are often more sensitive to immune checkpoint blockade therapy and therefore could serve as a prognostic indicator of clinical outcome, and could shed additional light on the mechanisms of tumorigenesis.

## Supplementary Material

Supplemental Material

## Data Availability

The data that support the findings of this study are openly available at NCBI GEO at https://www.ncbi.nlm.nih.gov/geo/query/acc.cgi?acc=GSE267317 (reference number GSE267317).
