# Supplementary material for: Fusobacterium nucleatum upregulates the immune inhibitory receptor PD-L1 in colorectal cancer cells via the activation of ALPK1
Source: Gut Microbes. 2025 Jan 29;17(1):2458203. doi: 10.1080/19490976.2025.2458203 (PMC11784648; doi:10.1080/19490976.2025.2458203)
Supplement: Supplemental Material [file KGMI_A_2458203_SM4676.zip › supplementary_files_2458203_1737520796479/Supplement figure legends.docx]

**Figure S1. Sodium periodate pre-treatment of HT-29 cells does not abrogate the respond to *F. nucleatum* supernatant**

HT-29 cells were mock-treated or pre-treated with sodium periodate for 1 h prior to the addition of cell-free bacterial conditioned culture medium from F. nucleatum 23726 or GMM, after which CXCL8 release after 24 h of stimulation was measured. Values represent mean ± SEM of three independent experiments performed in duplicate. A Ratio paired t test comparing stimulations to conditioned medium was used for statistical analysis. ns, not significant.

**Figure S2. A selection of intestinal commensal bacteria does not induce ALPK1-dependent NF-κB activation in HEK293 cells.** HEK293 wildtype cells and HEK293 ALPK1^-/-^ cells, transfected with an NF-κB luciferase reporter plasmid, were stimulated with TNF, ADP-heptose, bacterial culture medium or conditioned culture medium of various bacterial species. NF-κB activation was measured after 5.5 h stimulation and calculated as fold increase over the bacterial medium control. Values represent mean ± SEM of three independent experiments performed in triplicate. Unpaired t tests were used for statistical analysis of HEK293 cells compared to HEK293 ALPK1^-/-^ cells. ***p < 0.001.

**Figure S3. The impact of oxygen concentrations on the activation of HT-29.** HT-29 cells were treated with cell-free bacterial conditioned culture medium from F. nucleatum 23726, ADP-heptose, flagellin of none stimulation under atmospheric O_2_ concentrations (21%) or hypoxic conditions (5% O_2_), after which CXCL8 release after 24 h of stimulation was measured. Values represent mean ± SEM of three independent experiments performed in duplicate. A Ratio paired t test comparing stimulations to conditioned medium was used for statistical analysis. *, p < 0.05.

**Figure S4. Flagellin induces a pro-inflammatory signature in HT-29 cells.** A) Heatmap of the significantly up- and downregulated genes in HT-29 cells stimulated with flagellin compared to non-stimulated (No Stim) cells. B) Volcano plot of the up- and downregulated genes resulting from Flagellin stimulation, a number of highly upregulated genes is indicated. C) MSigDB Hallmark over-representation geneset analysis of top 5 up- and downregulated sets of genes. D) Gene Set Enrichment Analysis (GSEA) plots using MSigDB gene sets. The top half shows the Running Enrichment Score for the top 5 most differentially regulated gene sets (indicated by different colors), colored bars indicate the position of each of the members of the gene sets along the ranked gene list (Rank in Ordered Dataset). Bottom half shows the Ranked List Metric as bar plot (log2 fold change), which measures the degree of correlation of genes with the gene sets (left for positive and right for negative correlation).

**Figure S5. ADP-heptose does not induce transcriptional differences in healthy intestinal organoids.** A) Heatmap of the significantly up- and downregulated genes in healthy rectal organoids stimulated with flagellin compared to non-stimulated (No Stim) organoids. B) Volcano plot of the up- and downregulated genes resulting from Flagellin stimulation, a number of highly upregulated genes is indicated. C) Volcano plot of the up- and downregulated genes resulting from ADP-heptose stimulation. None of the genes showed significant alterations in regulation.

**Figure S6. ADP-heptose stimulation does not alter cell proliferation in HT-29 cells.** HT-29 cells and HT-29 *ALPK1*^-/-^ cells were stained with CellTrace^TM^ CFSE, stimulated with 500 ng/ml ADP-heptose or no stimulation, and proliferation was assessed after 24, 48 and 72 hours by median fluorescent intensity (MFI). Values represent mean ± SEM of three independent experiments. Unpaired t tests were used for statistical analysis of stimulated versus non-stimulated cells. No differences were observed between any of the compared conditions.

**Table S1. Bacterial strains used in this study.**

**Table S2. ADP-heptose-stimulated HT-29 DEG and ORA.**

Differentially expressed genes (DEG) in HT-29 cells after stimulation with ADP-heptose as calculated using DESeq2. Genes with a minimum fold-change of 2 with a false discovery rate of 0.05 as calculated by Benjamini-Hochberg correction are shown. Upregulated genes are shown in green, downregulated genes are shown in red. Differentially regulated pathways and genesets as calculated by MSigDB over-representation (ORA) analysis in HT-29 cells following stimulation with ADP-heptose. Genes with a minimum fold-change of 1 with a false discovery rate of 0.05 as calculated by Benjamini-Hochberg correction are included. Pathways and geneset with an adjusted p value of <0.05 are shown.

**Table S3. Flagellin-stimulated HT-29 DEG and ORA.**

Differentially expressed genes (DEG) in HT-29 cells after stimulation with flagellin as calculated using DESeq2. Genes with a minimum fold-change of 2 with a false discovery rate of 0.05 as calculated by Benjamini-Hochberg correction are shown. Upregulated genes are shown in green, downregulated genes are shown in red. Differentially regulated pathways and genesets as calculated by MSigDB over-representation (ORA) analysis in HT-29 cells following stimulation with flagellin. Genes with a minimum fold-change of 1 with a false discovery rate of 0.05 as calculated by Benjamini-Hochberg correction are included. Pathways and geneset with an adjusted p value of <0.05 are shown.
